# Molecular Characterization and Antimicrobial Resistance Pattern of *Escherichia coli* Recovered from Wastewater Treatment Plants in Eastern Cape South Africa

**DOI:** 10.3390/ijerph15061237

**Published:** 2018-06-12

**Authors:** Aboi Igwaran, Benson Chuks Iweriebor, Anthony Ifeanyi Okoh

**Affiliations:** 1 SAMRC Microbial Water Quality Monitory Center, University of Fort Hare, Alice 5700, South Africa; benvida2004@yahoo.com (B.C.I.); AOkoh@ufh.ac.za (A.I.O.); 2 Applied and Environmental Microbiology Research Group (AEMREG), Department of Biochemistry and Microbiology, University of Fort Hare, Private Bag X1314, Alice 5700, South Africa

**Keywords:** *Escherichia coli*, diarrheagenic, susceptibility, WWTPs

## Abstract

Wastewater treatment plants (WWTPs) are designed to eliminate organic matter and pathogens but most WWTPs discharges antimicrobial resistance pathogens into aquatic milieu. The study aimed to examine the antibiotics resistant patterns and the presence of some resistance genes among *E. coli* isolates from WWTPs effluents. Water were collected from WWTPs final effluents, filtered through nitrocellulose membrane and the filter papers were placed on chromogenic agar plates, incubated for 24 h at 37 °C. Presumptive *E. coli* isolates (173) were obtained from the culture method. From the presumptive *E. coli* isolates screened by polymerase chain reaction (PCR), 111 isolates were positive and the positive isolates were further screened for six diarrheagenic *E. coli* pathotypes (EPEC, ETEC, EHEC, DAEC, EIEC, and EAEC) and from the pathotypes screened, nine isolates harboured *daaE* gene. The phenotypic susceptibility patterns of the 111 isolates to 12 antibiotics were determined by Kirby-Bauer disk diffusion technique. All the isolates were resistant to erythromycin and clindamycin. From the resistance genes screened, 31 isolates harboured *mcr-1* gene and nine isolates harboured *ermA* gene. The study reveals that water samples recovered from the final effluents of WWTPs may likely be one of the major sources of antibiotic-resistant in *Escherichia coli*.

## 1. Introduction

Wastewater treatment plants (WWTPs) serve as a vehicle of antibiotic-resistance interface between the environment and the human society. Sewage from households, hospitals, or agricultural waste contains antibiotics-resistance bacterial of human and animal origin [1]. WWTPs are generally known as “hotspots” for the occurrence, development, and dissemination of antibiotics-resistance among bacteria which can acquire this resistance gene from donor to the recipient bacteria or through selective pressure influenced by residual antibiotics-resistant microbes [2]. When these antibiotic-resistant bacteria are not completely removed from the waste water treatment plants via treatment process, they will then be discharged into aquatic and the wider terrestrial environment which will eventually enter the human system through the food chain [3]. Wastewater treatment plants receive sewage from diverse sources and bacteria from this various environments interact and exchange antibiotic-resistance genes horizontally. Wastewater treatment plants (WWTPs) are hotspots for horizontal gene transfer in bacteria allowing the spread of antibiotic-resistance gene between different bacterial species [4]. The discharge of treated final effluent from WWTPs into streams, ponds, earth-dams, and canals is one of the major sources of potential pathogenic contamination in surface waters [5]. Most WWTPs are designed majorly to eliminate pathogens and organic matter [6], but WWTPs discharge pathogens harbouring antimicrobial resistance (AMR) determinants into the environment, resulting in horizontal gene transfer between bacterial and other pathogens which is a global health concern with the prediction of widespread untreatable infections within the next generation [7]. *Escherichia coli* is one of the most used indicator organisms to monitor the microbial quality of water [8]. Disease causing *E. coli* is grouped into intraintestinal *E. coli* causing diarrhea and extraintestinal *E. coli* which is responsible for a range of illnesses in humans and animals [9]. Among the bacterial pathogens, diarrheagenic *E. coli* is the main cause of globally epidemic and endemic diarrhea [10,11,12]. Diarrheagenic *E. coli* (DAE) strains are grouped into eight pathotypes which includes enteropathogenic *E. coli*, enterohaemorrhagic *E. coli*, enterotoxigenic *E. coli*, enterinvasive *E. coli*, and enteroaggregative *E. coli*, diffusely-adherent *E. coli*, diarrhea-associated hemolytic and cytolethal distending toxin producing *E. coli* [13]. Diarrheagenic *E. coli* harbour a virulence element that is responsible for causing disease in human [14,15]. *E. coli* has been reported to be highly resistance to some antibiotics used in the treatment of diseases [16]. The occurrence of antibiotic-resistant in pathogenic bacterial strains has great health implications such as longer hospitalization [17]. The emergence of multi-resistant Gram-negative bacteria against the existing antibiotics is disappointing; nonetheless, colistin is an old antibiotic that has been reported to be effective against multi-resistant Gram-negative bacteria but was dumped in human treatment due to its neurotoxicity and nephrotoxicity effect [18]. However, the increase in the emergence of multi-resistant bacterial strains has enforced clinicians to use colistin as one of the last-resort drugs of choice to fight infections [19]. 

South Africa has 986 municipal water-treatment facilities [20] and about 26% of sewage is inadequately treated before being discharged into rivers [21]. However, the high level of the occurrence of bacterial pathogens in streams and rivers poses a direct health risk to the people drawing water from these surface-water sources. Studying the antimicrobial susceptibility pattern of these bacterial strains (*E. coli*) is significant in order to identify the shift in antibiotic resistance patterns among these pathogens and to adopt control measures that will help in preventing the spread of multidrug-resistant or resistant strains of bacteria that will help to guard clinicians on antibiotics use [22]. To our best awareness, there are few studies on the incidence of diarrheagenic *E. coli* and their antibiotic susceptibility pattern recovered from WWTPs in Alice and Fort Beaufort, Easter Cape South Africa. The aims of this study was to isolate *E. coli* from final effluent of selected WWTPs, in Alice and Fort Beaufort, determine their antibiotic susceptibility pattern, delineation of the isolates into various *E. coli* pathotypes and screen for the presence of resistant genes using molecular approaches.

## 2. Materials and Methods 

### 2.1. Study Area and Collection of Water Samples

The study was conducted in Alice and Fort Beaufort with geographical coordinates of 32°47′0″ South, 26°50′0″ East, 32°76′63″ South, 26°62′00″ East, respectively, and both towns are located in Eastern Cape Province of South Africa. Water samples from the final effluent of the municipal WWTPs were collected once a week for three months. Samples were collected with sterile 1000 mL Nalgene bottles and were collected separately at three different points throughout the sampling period from the final effluents, transported on ice packs to the laboratory and processed upon arrival. The two WWTPs use activated sludge treatment technology and disinfect their final effluents by chlorination before discharging the sewage into the receiving water bodies. 

### 2.2. Bacterial Isolation

After shaking, each effluent sample of 100 mL from the 45 water samples analyzed were filtered through nitrocellulose membrane filters (0.45-μm pore size, Millipore, Durban, South Africa) adopting the membrane filtration technique. The membrane filter papers were picked with a sterilized forceps, placed onto chromogenic agar plates and the plates were incubated for 24 h at 37 °C. After overnight incubation, 173 presumptive colonies of *E. coli* were picked, streaked onto nutrient agar plates and incubated for 24 h at 37 °C. Pure colonies from the nutrient agar plates were inoculated into 7 mL of Luria-Bertani broth, incubated for 24 h at 37 °C and from which glycerol stocks were prepared and stored at −80 °C for future analyses.

### 2.3. Extraction of Bacterial DNA

Bacterial DNA was extracted by boiling method as described by Momtaz et al. [23] with little modification. Presumptive *E. coli* in glycerol stocks stored at −80 °C were resuscitated in Luria-Bertani broth, incubated for 24 h at 37 °C followed by DNA extraction as follows. For the bacteria DNA extraction, 200 µL of the overnight broth was transfer into DNase free 2 mL microcentrifuge tubes, centrifuged at 15,000 rpm for 10 min and the supernatant was decanted. The pellet was re-suspended in 200 µL sterile distilled water; vortexed and the cells were lysed by boiling on AccuBlock (Digital dry bath, Labnet, Staffordshire, UK) for 15 min at 100 °C and thereafter centrifuge at 15,000 rmp for 5 min. DNA-containing supernatants were transferred into another DNase free microcentrifuge tubes and were stored at −20 °C for future analyses.

### 2.4. Molecular Confirmation of E. coli and E. coli Pathotypes

Presumptive *E. coli* isolates were confirmed by polymerase chain reaction (PCR) targeting the *uidA* gene as earlier described by Janezic et al. [24]. The pathotypes of the confirmed *E. coli* isolates were determined by PCR technique making use of specific primers targeting *virF* gene for enteroinversive *E. coli* (EIEC), *aafII* gene for enteroaggregative *E. coli* (EAEC), *daaE* gene for diffusely adherent *E. coli* (DAEC), *eae* gene for enteropathogenic *E. coli* (EPEC), *stx1* gene for enterohaemorrhagic *E. coli* (EHEC) and *stII* gene for enterotoxigenic *E. coli* (ETEC) as listed in Table 1. Verification of amplification of the PCR products of various reaction mixtures was carried out by resolving them in a 1.5% agarose gel electrophoresis for 35 min at 120 Volts in 0.5% TBE buffer stained with ethidium bromide solution. The resolved PCR products were visualized and photographed under UV light trans-illuminator (ALLIANCE 4.7) molecular Imager Gel Doc system. 

### 2.5. Antimicrobial Susceptibility Testing

Antimicrobial susceptibility testing of the confirm *E. coli* isolates was determined by disc diffusion method on Muller-Hinton agar (MHA) plates following Clinical and Laboratory Standard Institute (CLSI) [27] guidelines. Fresh culture from the glycerol stock was streaked on nutrient agar plates, incubated at 37 °C for 24 h. Colonies were transferred into test tube of 5 mL of normal sterile saline adjusted to obtain turbidity matching 0.5 Mc-Farland standards. The isolates were inoculated onto MHA plates and disks impregnated with antimicrobial agents were dispensed on the inoculated plates, incubated at 37 °C for 18–24 h and zones of inhibition were measured after the incubation periods. Each isolate was classified as resistant or susceptible to antimicrobial agents used while those that were intermediate were considered resistant. The commercial antibiotic discs used were: Amoxicillin (25 µg), Cefuroxime (30 µg), Gentamicin (10 µg), Doxycycline (30 µg), Ciprofloxacin (5 µg), ofloxacin (5 µg), Trimithoprime (5 µg), Menopenem (10 µg), Colistin-Sulphate (10 µg), Erythromycin (15 µg), Clindamycin (2 µg) and Sulphamethoxazole (25 µg). Table 2 show the names, concentrations and interpretation of the antibiotic discs used.

## 3. Results

### 3.1. Molecular Confirmation of E. coli Isolates

**Figure 1:**
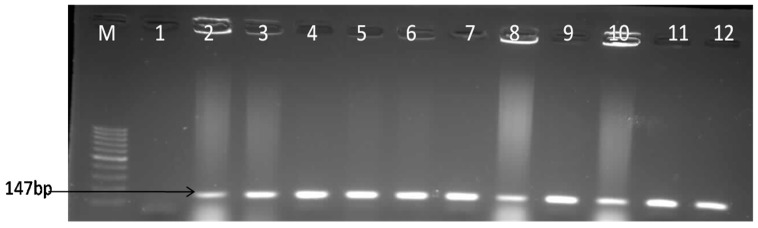
Gel image for the molecular confirmation of *E. coli* isolates using the *uidA* (147bp) gene. Lane M: 100bp DNA ladder, Lane 1: Negative control, Lane 2–12 samples.

The occurrences of 173 *E. coli* isolates were detected in water samples collected from the three different sampled points of the final effluent of the WWTPs and PCR analysis confirmed these findings. Among the 173 isolates screened, 111 (64.16%) were confirmed positive for *E. coli* targeting the housekeeping *uidA* gene. Figure 1 show the confirmed molecular gel image of the amplified product of *uidA* (147bp) of some of the identified positive *E. coli* isolates.

### 3.2. Molecular Confirmation of E. coli Pathotypes

Among the six *E. coli* pathotypes screened for by PCR technique with specific oligonucleotide primers targeting each of this diarrheagenic *E. coli*, only nine (8.1%) were positive for diffusely adherent *E. coli* (DAEC) that harboured *daaE* gene while none was positive for EIEC, EPEC, EAEC, ETEC, and EHEC and the result is as shown in Table 3. Among the nine *E. coli* isolates that harboured *daaE* gene, only one isolate was susceptible to CIP, MEM, CXM, and GM while the rest isolates were all resistance to all the antibiotics. 

### 3.3. Antimicrobial Susceptibility Pattern of the Confirmed E. coli Isolates

**Figure 2:**
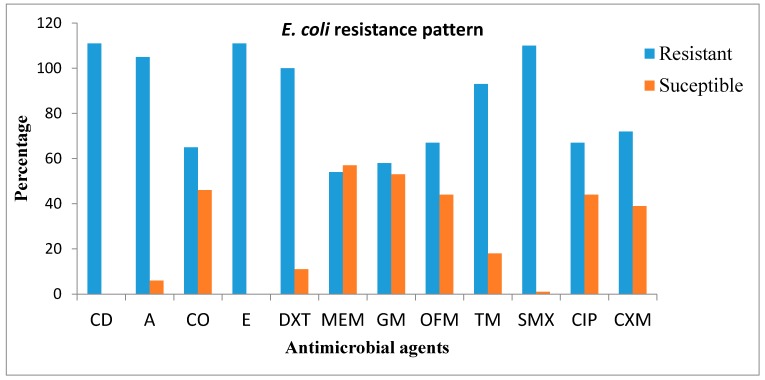
Antimicrobial susceptibility patterns of *E. coli* isolated from wastewater treatment plants (WWTPs) in the Eastern Cape South Africa. Clindamycin (CD), Amoxicillin (A), Colistin-sulphate (CO), Erythromycin (E), Doxycyline (DXT), Cefuroxime (CXM), Meropenem (MEM), Gentamicin (GM), Ofloxacin (OFX), Trimethgoprim (TM), Sulphamethaxole (SMX), and Ciprofloxacin (CIP).

Antibiotic susceptibility pattern of the isolates tested against various antibiotics following Clinical and laboratory standard Institute, (CLSI) [27] Guidelines showed that clindamycin and erythromycin (100% each) had the highest percentage resistance. The following is the frequency of the level of resistance exhibited by *E. coli* isolates against the antibiotics tested; clindamycin and erythromycin (100% each), sulphamethoxazole (99%), amoxicilin (94.5%), doxycycline (90%), trimithoprime (83.7%), cefuroxime (64.8%), ofloxacin and ciprofloxacin (60.3% each), colistin-sulphate (58.5.1%), gentamicin (52.2%), and menopenem (48.6%) and those that showed intermediate were considered as resistant. The antimicrobial susceptibility patterns of the isolates recovered from WWTPs in the Eastern Cape Province of South Africa are shown in Figure 2.

### 3.4. Resistance Determinants among the Isolates

The resistant genes screened by molecular technique were *ermA* and *mcr-1*. Among the 65 *E. coli* isolates that showed phenotypic resistance to colistin as shown in Figure 2, 31 isolates harboured *mcr-1* gene, and the 111 isolates that showed phenotypic resistance to erythromycin, only nine isolates harboured *ermA* gene. Table 4 showed the number of confirmed *E. coli* isolates recovered from WWTPs that were resistance to *ermA* and *mcr-1* of genes. The choice for screening for the presence of these resistance genes is because of the high phenotypic resistance in *E. coli* isolates to erythromycin and also to give an update on the efficacy of the last-resort (colistin) antibiotics.

## 4. Discussion

The final effluent of wastewater treatment plants is a major vehicle of antimicrobial resistant pathogens into the aquatic environment. However, the aquatic environment also helps in the spread of antibiotic resistance genes through transfer of genetic material among these pathogens [30]. Microbiological water quality standards are majorly based on faecal indicators; though they signify a minor part of the total bacterial in aquatic environment and *E. coli* is a frequently used indicator organism used to monitor the microbial quality of water [31]. From the 173 presumptive *E. coli* isolates screened, 111 isolates harboured *uidA* gene through molecular technique. This result highlights the high occurrence of *E. coli* isolates recovered from the final effluent of the two WWTPs and this water being discharged into the environment poses a serious threat to public health and this is in support with the report of Osińska et al. [32,33]. *E. coli* contamination of water is, and remains a regular and persistent problem, impacting negatively on human health and national economies [34]. Diarrheagenic *E. coli* are the principal cause of death globally especially in developing countries where about 1.6–2.5 million deaths occur yearly as a result of diarrhea [13,35]. Among the six diarrheagenic *E. coli* strains profiled for from the 111 confirmed *E. coli* isolates, only nine (8.1%) isolates harboured *daaE* gene while none was positive for EIEC, EPEC, EAEC, ETEC, and EHEC. This implies that WWTPs serves as reservoir of some diarrheagenic *E. coli* pathotypes and this is in agreement with the report of Mokracka et al. [36]. Study of Omar & Barnard [37] also detected the presence of diarrheagenic *E. coli* from the final effluent of wastewater treatment plant and this also corroborated our findings. Another study of Sidhu et al. [38] also identified the presence of some of these diarrheagenic *E. coli* strains in surface water and this accentuate the global occurrence of potential diarrheagenic *E. coli* pathotypes in the aquatic environment that poses high risk of waterborne infections. Infections caused by these diarrheagenic *E. coli* strains are treated with antibiotics and the effectiveness of these antibiotics in the treatment of these infections is being compromised due to the increasingly emergence of resistant strains to most first-line antimicrobial agents [39]. 

Antimicrobial susceptibility testing is a well-known global standard enabling laboratories to help clinicians in treating infections caused by bacteria [40]. Antibiotics are commonly used therapeutic agents against infections caused by pathogenic bacterial strains; however, increase in resistance in these bacterial strains to these antibiotics need urgent actions to curtail the spread of these resistant microorganisms that poses major threats to human and animal health [41]. From the confirmed *E. coli* isolates that were tested against a panel of 12 commercial antimicrobial agents, the isolates exhibited different phenotypic resistance patterns against the antimicrobial agents ranging from CD and E (100% each), SMX (99%), A (94.5%), DXT (90%), TM (83.7%), CXT (64.8%), OFX and CIP (60.3% each), CO (58.5.1%), GM (52.2%), and MEM (48.6%). All the isolates were resistance to CD and E and also exhibited resistance to a wide range of antimicrobial agents as shown in Figure 2. The occurrence of antibiotic-resistant bacteria in the environment poses a serious threat to public health, adds to higher disease burden, reduces the effectiveness of these antibiotics and increases the mortality rate [33]. Li et al. [42] also reported high resistance rate in *E. coli* isolates recovered from pig faecal samples and our finding is accordance with their report. The study of Adwan et al. [43] also revealed high resistance rates (95%) in *E. coli* isolate to erythromycin and this also corroborated with our result. 

The aquatic milieu appears to allow antibiotic resistance genes of sewage origin to continue and spread into the environment which therefore increases the high risk of gene transfer to human and animal through food chains [44]; injection of contaminated water or through direct contact with infected person [45]. WWTP final effluents used as irrigation water can promote the distribution of antibiotic resistance genes into soils or water that somehow could find their ways into human system [46]. The detection of *erm* resistance genes by molecular method has regularly been used to monitor the loads of antibiotic resistance gene in different environments [47]. However, genetic screening of the 111 *E. coli* isolates that showed phenotypic resistance to erythromycin (resistance and intermediate), only nine (8.1%) isolates harboured *ermA* gene and the result of the phenotypic resistance gene do not correspond with the phenotypic resistance as shown in 2. Ziembińska-Buczyńska et al. [48] and Yuan et al. [49] also reported the presence of erythromycin phenotypic resistance gene (*erm* gene) in *E. coli* recovered from wastewater treatment plant and their report is in line with our result. From the antimicrobial susceptibility test result shown in Figure 2, 65 confirmed *E. coli* isolates demonstrated phenotypic resistance to colistin and when these 65 *E. coli* isolates were screened by the PCR technique, 31 (47.6%) isolates harbours *mcr-1* gene as showed in Table 4. Colistin sulphate is regarded as a last-resort antibiotic against multi-resistant bacterial pathogens as reported by Curcio et al. [50] and the finding from this study revealed high occurrence of colistin-resistance (*mcr-1*) gene which could be higher in the communities serviced by the WWTPs under investigation. *E. coli* developing resistance to colistin is usually associated with chromosomal mutations [51]; however, a new plasmid-mediated conveyable resistance determinant which is *mcr*-*1* gene encoding for phosphoethanolamine transferase has been established as a marker for the detection of colistin resistance [29]. Quesada et al. [52] and Islam et al. [53], also reported colistin resistance *E. coli* isolates carrying *mcr-1* gene and this is in concordance with our result. The continued spread of *mcr-1* gene in *E. coli* and other bacterial strains will compromise the clinical usefulness of last-resort antibiotic which could result to additional antibiotic treatment failure and extensive morbidity and mortality [54]. One of the mechanism in which these bacterial that harboured these resistance genes can be transferred to human is through consumption of contaminated foods from animal origin [55,56]. All the isolates that showed phenotypic resistance to the test antmicrobial agents do not correspond with the number of confirmed resistance genes and this could be attributed to the fact that there are several variants of resistance determinants responsible for the observed phenotypic resistances among the isolates [57].

## 5. Conclusions

During the treatment process of WWTPs, microbes are notably reduced but vast quantities of antimicrobial agents as well as drug-resistant bacteria escaping the treatment process are channeled into aquatic milieu with treated effluents. The discharge of these treated effluents into the aquatic environment could possible increase antibiotic-resistance in *E. coli* thus escalating the spread of drug-resistant microbes in communities using the streams and water bodies receiving the treated effluent. The high prevalence of colistin-resistance gene in *E. coli* recovered from the final effluent pose a high risk to the people in this study area that depend on this surface water bodies as their major source of water and this is the first report on colistin-resistance *E. coli* carrying the *mcr-1* gene.

## Figures and Tables

**Table 1:** Primers sets for *uidA* gene of *E. coli* and various genes of *E. coli* pathotypes.

Primer Sequence (5′–3′)	*E. coli* and Pathotypes	Targeted Genes	Base Pair	References
F-GAACGTTGGTTAATGTGGGGTAA	*E. coli*	*uidA*	147	[25]
R-ACGCGTGGTTACAGTCTTGCG				
F-GAACGTTGGTTAATGTGGGGTAA	*DAEC*	*daaE*	542	[26]
R-TATTCACCGGTCGGTTATCAGT				
F-AGCTCAGGCAATGAAACTTTGAC	*EIEC*	*virF*	618	[26]
R-TGGGCTTGATATTCCGATAAGTC				
F-CACAGGCAACTGAAATAAGTCTGG	*EAEC*	*aafII*	378	[26]
R-ATTCCCATGATGTCAAGCACTTC				
F-TCAATGCAGTTCCGT TATCAGTT	*EPEC*	*eae*	482	[26]
R-GTAAAGTCCGTTACCCCAACCTG				
F-CAGTTAATGTGGTGGCGAAGG	*EHEC*	*stx1*	348	[26]
R-CACCAGACAATGTAACCGCTG				
F-GCACACGGAGCTCCTCAGTC	*ETEC*	*stII*	129	[26]

**Table 2:** Primers sets used in targeting various resistance genes.

Primer Sequence (5′–3′)	Targeted Genes	Base Pair (bp)	Reference
F-GTTCAAGAACAATCAATACA GAG	*ermA*	421	[28]
R-GGATCAGGAAAAGGACATTT TAC			
F-CGGTCAGTCCGTTTGTTC			
R-CTTGGTCGGTCTGTAGGG	*Mcr-1*	309	[29]

**Table 3:** Number of confirmed *E. coli* Pathotypes.

*E. coli* Pathotypes	DAEC	EIEC	EPEC	EAEC	ETEC	EHEC
Targeted genes	*daaE*	*virF*	*eae*	*aafII*	*stII*	*stx1*
No of positive isolates	9	0	0	0	0	0

**Table 4:** Numbers of confirmed *E. coli* isolates resistant to *ermA* and *mcr-1* genes.

Targeted Genes Screened	Total No of Isolates Screened	No of Isolates That Showed Phenotypic Resistance to the Text Antibiotic	No of Confirmed Isolates
*ermA*	111	111	9
*mcr-1*	111	65	31
